# Prevalence of influenza A/H3N2 virus in northern Iran from 2011 to 2013

**Published:** 2015

**Authors:** Mohammadreza Haghshenas, Elham Jafarian, Farhang Babamahmoodi, Ahmad Tabrizi, Sharbano Nandoost, Reza Alizadeh-Navaei

**Affiliations:** 1 Molecular and Cell Biology Research Center, Faculty of Medicine, Mazandaran University of Medical Sciences, Sari, Iran.; 2 Student Research Committee, Mazandaran University of Medical Sciences, Sari, Iran.; 3 Antimicrobial Resistance Research Center, Faculty of Medicine, Mazandaran University of Medical Sciences, Sari, Iran.

**Keywords:** Influenza A, Influenza A/H3N2, Swine flu, Season flu

## Abstract

**Background:**

Influenza A virus is the most virulent human pathogen and causes the most serious problem. Having epidemiological knowledge about this disease is important. The aim of this study was to determine the prevalence of influenza A/H3N2 virus infection in northern Iran from 2011 to 2013 using the real-time polymerase chain reaction (RT-PCR).

**Methods:**

In this cross-sectional study 57 samples were collected from patients with influenza-like illness (T≥ 38 °C and cough or sore throat. Influenza-RNA was extracted from the samples using PureLink^TM ^Viral RNA/DNA Kit. RT-PCR was one using SuperScript III Platinum, Quantitive Real Time PCR system from invitrogen with a specific type of primers and probs. All samples were examined in the Influenza laboratory of Mazandaran University of Medical Sciences.

**Results:**

The mean age of patients was 38.2±14.5 year, 278 (48.69%) were males and 293 (51.31%) females. A total number of 201 patients (35.2%) were diagnosed as influenza A1 H3 N2 infection.

**Conclusion:**

The results show that the prevalence of A/H3N2 in the North of Iran is considerable and needs more attention for preventive measures.

Influenza infection is one of the most prominent respiratory infections of human being which causes severe morbidity and mortality every winter (1, 2). Influenza viruses are classified into three types; influenza A, influenza B and influenza C virus (3), with relevance to humans for each type. Type A is the most popular and serious human pathogen among the three types (4), and is associated with seasonal epidemics in temperate regions (2, 5, 6). Influenza A virus may infect different animal hosts including birds, horses, sea mammalians, pigs and humans (3, 7). Influenza annually spreads around the world in seasonal epidemics, and is associated with high mortality estimating to affect 5-15% of the global population (8-10). The diagnosis of influenza can be made clinically in appropriate settings. Infection with influenza presents as a spectrum of symptoms ranging from patients who are asymptomatic to those with fulminant primary viral pneumonia, depending on host immune status, type and the dose of virus (11, 12). The methods currently used for the diagnosis of influenza include direct immunofluorescence, enzyme-linked immunosorbent assays, serologic testing, viral culture and polymerase chain reaction (PCR). PCR has proven to be more sensitive and more specific than traditional culture techniques as well as other tests for the detection of influenza viruses (13, 14). The aim of this study was to investigate the epidemiology of influenza A1 H3 N2 infections in patients presented to hospitals of northern Iran from 2011 to 2013 diagnosed by real time PCR (RT-PCR).

## Methods

**Samples: **This study was a cross-sectional survey performed between 2011 and 2013 on 571 patients with influenza-like illness (T≥ 38 °C and cough or sore throat) in the North of Iran, Mazandaran. Sample size was calculated based on a=0.05, P=0.4 [according to Hanshaoworakul et al.’s study (15)], d=0.04 and Cochran formula. Specimen samples for testing were typically sputum, swabs of the throat and washes, and swabs of the nasopharyngeal. Common cold samples were collected from patients and influenza-RNA was extracted from samples. Aliquots of material were made immediately upon receiving the specimens and either tested immediately or stored at -70°C until use. Factors such as, age, gender, suspected sources of infection were chosen through a questionnaire for all patients. Data were analyzed with SPSS 15 software. 

**RNA extraction: **RNA was extracted from clinical specimens of the throat or nasopharyngeal by PureLink^TM^ Viral RNA/DNA Kit (Invitrogen) using the standard protocol. Influenza A/H3N2 was isolated from specimen samples on following procedures; 25µl proteinase K was added into ependorph tube and 200µl specimen samples of patients with 200 µl lysis buffer were mixed by vortex at 15 seconds and incubated for 15 minutes at 56°C, and fast centrifuged. 250 µl of 96%-100% alcohol was added into tubes and mixed by vortex at 15 seconds, and incubated for 15 minutes at 56°C. 675 µl of previous samples were transfered into Viral Spin C tubes and centrifuged for one minute at 12000 revolutions per minute. The Viral Spin C filter was moved into a new sterile microtube and washed two times with 500 µl of Wash buffer, and centrifuged for one minute at 12000 revolutions per minute, and then centrifuged for one minute at high speed. Next, the Viral Spin C filter was moved into a new sterile 1.5 ml microtube and added 50 µl of distil water or RNase free water in the middle of the Viral Spin C filter, and incubated at room temperature for one minute and then centrifuged for one minute at high speed. Finally, RNA quantification was determined using a spectrophotometer and resulted residue solved for next stages or stored at -70°C until use. All samples were examined at the Influenza laboratory of Mazandaran University of Medical Sciences.

**Real Time PCR: **Total RNA was isolated from samples and RT-PCR was done using the SuperScript III Platinum, Quantitive Real-time PCR system Kits (invitrogen) according to distinc protocol with primers and independent probes (see table 1). Primers and probes, after diluting, stored in -20^°^C; and all steps of preparing reactional mix over ice resells were performed. Examination method summarized as follows: 2x reaction Mix 10µl, forward primer (40 UM) 0.4µl, reverse primer (40 UM) 0.4µl, probe (10UM) 0.4µl, Super Script III RT/ Platinum- Taq mix 0.4µl, RNase – DNase Free water 5.4µl, aggregation was 17µl. 16 µl of the prior reaction mix extracted with 4 µl of RNA extraction samples, final reaction volume was 20 µl. The samples were placed into the 96-well real time RT-PCR plate and amplified. Amplification was proceeded at 60°C for 30 minutes, 95°C for five minutes and then for 45 cycles of 95°C for 15 seconds, 55°C for 30 seconds and 72°C for 30 seconds. PCR reaction was performed using a CFX96 Real-time system (Bio-Rad) sequence detector. After this stage, using a computer program, immediately the results were determined in programming RT–PCR, besides the positive test, amount and quantity of virus was determined. 

**Table 1:** Sequence of primers and probes of influenza A and A/H3N2 viruses

**Sequence**	**Primers & Probes**
GAC CRA TCC TGT CAC CTC TGA C	Inf A Forward
AGG GCA TTY TGG ACA AAK CGT CTA	Inf A Reverse
TGC AGT CCT CGC TCA CTG GGC ACG	Inf A Probe
AAG CAT TCC YAA TGA CAA ACC	S W H3 Forward
ATT GCR CCR AAT ATG CCT CTA GT	S W H3 Reverse
CAG GAT CAC ATA TGG GSC CTG TCC CAG	S W H3 Probe

## Results

In the current study, 571 sputum samples, swabs of the throat or nasopharyngeal from patients who referred to hospitals in the North of Iran were collected between 2011 and 2013. The mean age of patients was 38.2±13.4 (from two months to 84) years in which 278 (48.69%) patients were males and 293 (51.31%) were females. These sputum samples from patients were collected from the different cities in the North of Iran (Mazandaran). Suspect influenza cases received between 2011 and 2013 and tested by RT-PCR totaled 571; 352 (61.65%) cases were negative of clinical samples and 219 (38.35%) of clinical samples were obtained influenza viruses. In this research, for positive influenza viruses, 201 (35.20%) were obtained influenza A/H3N2 (CI95%: 31.2-39.1). In total cases were positive for influenza viruses, influenza A/H3N2-RNA was found in extremely high of samples.

In this study, the most prevalence of clinical samples from patients were collected between 21 and 30 years (25.57%) that influenza A/H3N2-RNA virus was detected 50% of clinical samples of that age group. In total, 37.5% of clinical samples that detected influenza A/H3N2-RNA, were between 21 and 30 years but in the clinical samples of over 61 years and children were obtained lower (table 2). 

**Table 2:** Influenza A/H3N2 virus distribution according to age group

**Age**	**Number of Flu symptom (%)**	**Number of A/H3N2 positive in group (%)**	**A/H3N2 positive in total positive patients (%)**	**A/H3N2 positive in total samples (%)**
10 and less than 10	56 (9.38)	21 (37.50)	10.44	3.67
20- 11	58 (10.16)	18 (31.03)	8.96	3.15
30- 21	146 (25.57)	73 (50.00)	36.32	12.78
40- 31	68 (11.91)	27 (29.71)	13.43	4.73
50- 41	60 (10.51)	20 (33.34)	9.95	3.50
60- 51	48 (8.41)	18 (37.50)	8.96	3.15
70- 61	73 (12.78)	16 (21.92)	7.96	2.80
0vere 70	62 (10.86)	11 (17.74)	5.74	1.93
Total or Average	100) 571)	201 (35.20)	100	35.2

## Discussion

In this study, we detected influenza A. We found it more prevalent in age group of 21-30 years old which accounted 50% of cases who presented with clinical features compatible with influenza infection. However, in older patients and children with similar clinical symptoms, the rate of infection was lower.

During 2004-2006 in Thailand, it has been reported that 2075 (18%) of clinical samples from patients were isolated human influenza viruses. In this report, 22 influenza related deaths were identified and eight of them were conformed influenza A/H3N2 virus, this was different from our result. It was shown that subtype A/H3N2 accounted for 38% of all human influenza infections in 2004 that was equivalent to the current study; for 50% of all infections human influenza in 2005 was more in this study, and less in 20% of all human influenza infections in 2006 that was less than the prevalence study (15). During 1994-1996, 2001-2006 and 2008, in Denmark, it was reported that the most of human influenza viruses in clinical samples was identified subtype A/H3N2 but during 2000, 2007 and 2009 similar to our study, the most of human influenza viruses in clinical samples was identified subtype A/H1N1 which was different from our study (16). During 2008, 932 clinical samples were collected from patients in Uganda, most of them (77%) were <5 years which were not similar to our research results. The rate of human influenza A virus was identified 72 (7.9%) of clinical samples from patients in which majority (52 clinical samples from patients) of them were confirmed influenza A/H3N2 virus (17). Result suggested that during a period of influenza outbreak in the community, the clinicians had a low threshold for suspecting, diagnosing, and treating the infection according to the recommended national guidelines. This study showed a high correlation between clinical findings and real-time PCR detection for the diagnosis of influenza virus infections. Seasonal human influenza virus is slighty different from one of last year’s flue season virus variants. To provide immune protection against human influenza viruses, vaccines of live attenuated viruses should be developed in the following years with wild-type and recombinant virus strains.

In limitation, this survey is a cross-sectional study and patients were not followed-up. In conclusion, the findings of this study indicate that a high proportion of patients presenting with clinical symptoms of influenza infection in North of Iran may have influenza H/H3N23 virus which can be diagnosed by PT-PCR .The results of this study provide a rational for preventive measures during the outbreak of influenza infection.

## Notes

**Funding:** This study was supported by the Vice-Chancellery for Research of Mazandaran University of Medical Sciences (Grant Number: 90-95).
